# The C5a/C5aR1 Axis Contributes to the Pathogenesis of Acute Cystitis Through Enhancement of Adhesion and Colonization of Uropathogenic *E. coli*

**DOI:** 10.3389/fcimb.2022.824505

**Published:** 2022-03-30

**Authors:** Kun-Yi Wu, Bo Cao, Chun-Xuan Wang, Xue-Ling Yang, Shu-Juan Zhao, Teng-Yue Diao, Li-Rong Lin, Guo-Xiu Zhao, Wuding Zhou, Ju-Rong Yang, Ke Li

**Affiliations:** ^1^ Core Research Laboratory, The Second Affiliated Hospital, School of Medicine, Xi’an Jiaotong University, Xi’an, China; ^2^ Department of Nephrology, The Third Affiliated Hospital of Chongqing Medical University, Chongqing, China; ^3^ Peter Gorer Department of Immunobiology, School of Immunology & Microbial Sciences, Faculty of Life Sciences & Medicine, King’s College London, London, United Kingdom

**Keywords:** urinary tract infection, acute cystitis, C5a/C5aR1, bladder inflammation, bacterial adhesion

## Abstract

Our previous work using a murine model of pyelonephritis demonstrated that the C5a/C5aR1 axis plays a pathogenic role in acute kidney infection. In this study, we report that the C5a/C5aR1 axis also plays a pathogenic role in acute bladder infection. C5aR1-deficient mice had reduced bladder bacterial load and attenuated bladder tissue injury, which is associated with reduced expression of terminal α-mannosyl residues (Man) (a potential ligand for type 1 fimbriae of *E. coli*) at the luminal surface of the bladder epithelium and reduced early bacterial colonization of the bladder. *In vitro*, C5a stimulation enhanced mannose expression in and facilitated bacterial adhesion/colonization to human bladder epithelial cells. C5a stimulation also upregulated the activation of ERK1/2 and NF-κB signaling and gene expression of proinflammatory cytokines (i.e., *Il6, Il1b, Cxcl1, Ccl2*) in the epithelial cells, which could drive pro-inflammatory responses leading to tissue injury. Administration of the C5aR1 antagonist effectively reduced bladder bacterial load and tissue injury. Thus, our findings demonstrate a previously unknown pathogenic role for the C5a/C5aR1 axis in bladder infection and suggest that the C5a/C5aR1 axis-mediated upregulation of Man expression, enhancement of bacterial adhesion/colonization, and excessive inflammatory responses contribute to acute bladder infection. These findings improve our understanding of the pathogenesis of bladder infection with therapeutic implications for UTI.

## Introduction

Urinary tract infections (UTIs) are among the most common infectious diseases, estimated to affect 40%–50% of women at least once in their lifetime (Lacerda Mariano and Ingersoll, 2020). Although antibiotics are available to treat the disease, there remain significant problems including frequent recurrence, persistence of infection, and the increasing risk of resistance to antibiotics (Wagenlehner et al., 2020). Virulent multidrug-resistant UPEC strains have recently been reported globally (Gupta and Bhadelia, 2014; Bunduki et al., 2021), which has significant impact on overall antimicrobial resistance. For these reasons, it is imperative to improve our understanding of the pathogenesis of UTI and develop novel treatment strategies that could be used to improve current treatment.

UTI is a wide spectrum of diseases including bladder infection (cystitis), kidney infection (pyelonephritis), and systemic infection with multiorgan failure (in severe case) (Ambite et al., 2021). UTI usually starts in the bladder and can spread to the kidney (Nielubowicz and Mobley, 2010; Klein and Hultgren, 2020). The majority of UTI (>80%) are caused by UPEC (Staerk et al., 2016). The pathogenesis of UTI can be influenced by both properties of the infecting pathogens and host responses to pathogens, in addition to other factors such as anatomical abnormality (Nielubowicz and Mobley, 2010; Abraham and Miao, 2015; Klein and Hultgren, 2020; Wagenlehner et al., 2020). Epidemiological studies have shown that >90% of all UPEC express type 1 fimbriae (Tchesnokova et al., 2011). Type 1 fimbriated *E. coli* are mostly associated with colonization of the lower urinary tract where they bind to uroplakins Ia and Ib, two major high mannose-type glycoproteins that coat on the apical surface of bladder epithelium (Wu et al., 2009; Staerk et al., 2016). Upon contact with uroepithelial cells, UPEC liberate toxins (e.g., hemolysin and cytotoxic necrotizing factor-1) (Ulett et al., 2013; Diabate et al., 2015) which mediate direct injury to the cells, disrupting the mucosal barrier and opening access to the underlying tissue. UPEC are also able to invade, survive, and multiply within bladder epithelial cells, which is thought to enable organisms to evade host defenses and act as a reservoir for further infection (Mulvey et al., 2001; Klein and Hultgren, 2020; Munoz et al., 2022).

Once UPEC establish contact with the urinary tract mucosa, rapid and robust host immune responses are triggered. Uroepithelial cells are usually the first to respond to microbial challenge and then orchestrate the subsequent host response by release of cytokines, other mediators of inflammation, and complement proteins and stimulate the influx of neutrophils (Spencer et al., 2014; Jones-Freeman et al., 2021). However, most human UPEC strains are resistant to complement-mediated killing (Li et al., 2009; Bjanes and Nizet, 2021), and several lines of study have suggested that these bacteria mediate excessive inflammatory responses that cause uroepithelial destruction (Wullt et al., 2003; Ambite et al., 2021; Paludan et al., 2021), impair innate immune cell function, and lead to chronic and recurrent UTI (Hannan et al., 2010; Choudhry et al., 2016).

C5aR1 is an N-linked glycosylated G protein-coupled receptor, which is expressed in myeloid cells and non-myeloid cells (e.g., endothelial and epithelial cells) (Reis et al., 2019). C5a and C5aR1 interactions mediate a broad spectrum of pro-inflammatory reactions (such as chemotaxis of leukocytes, histamine release, and generation of pro-inflammatory mediators). Previous studies have shown that C5a/C5aR1 interactions can contribute to the pathogenesis of a wide range of inflammatory and immunological diseases (e.g., ischaemia/reperfusion injury, sepsis, autoimmune disease, transplant rejection), suggesting that C5aR1-mediated excessive inflammatory response is an important pathogenic factor in those disorders (Rittirsch et al., 2008; Hashimoto et al., 2010; Peng et al., 2012; Stegall et al., 2012). In the context of infectious diseases, our recent study in a murine model of acute pyelonephritis has shown that the C5a/C5aR1 axis plays a pathogenic role in kidney infection (Choudhry et al., 2016; Li et al., 2017). It has also been suggested that the C5a/C5aR1 axis could contribute to kidney infection through two pathways: i) mediating excessive inflammatory responses and ii) upregulating the expression of the mannosyl residue (Man) (the potential ligand for type 1 fimbriae of UPEC) on the luminal surface of the renal tubular epithelium which promotes UPEC adhesion/colonization in the kidney (Li et al., 2017; Song et al., 2018). So far, it has been unknown whether the C5a/C5aR1 axis plays important roles in bladder infection; we therefore investigated this subject in the present study.

To this end, an established murine model of acute cystitis combining deletion of the *C5ar1* gene and blockade of C5aR1 with the C5aR1 antagonist was used to determine the role of C5aR1 in bladder infection. Bacterial load, bladder histopathological changes, neutrophil infiltration, and tissue inflammatory mediator expression were assessed following bladder inoculation with UPEC. The human bladder epithelial cell line (J82 cell) was used to examine the influence of the C5a/C5aR1 axis on Man expression and facilitated bacterial adhesion/colonization to uroepithelial cells.

## Materials and Methods

### Materials

The following reagents were used in this study: tryptone, yeast extract, cystine lactose electrolyte deficient (CLED) agar (Oxoid, Basingstoke, UK); monoclonal rat anti-mouse Ly6G, polyclonal goat anti-mouse C5aR1 (M-19) (Santa Cruz Biotechnology, Heidelberg, Germany); polyclonal rabbit anti-mouse cytokeratin (Abcam); Alexa Fluor 488 donkey anti-goat IgG (Jackson Immunology Research Lab., West Grove, USA); fluorescein-labeled *Galanthus nivalis* lectin (GNL, which binds to mannosyl residues) (Vector Laboratories, Peterborough, UK); polyclonal rabbit anti-human ZO-1 and 4′,6-diamidino-2-phenylindole (DAPI) (Life Technologies Ltd., Beijing, China); protease inhibitor cocktail, tetramethylrhodamine (TRITC) (Sigma-Aldrich, St. Louis, USA); anti-phospho-ERK1/2 (Thr202/Tyr204), -IκB (Ser 32), and anti-ERK1/2, -IκB antibodies used for Western blot and signaling pathway studies (Cell Signaling Technology, Danvers, USA); cell culture medium, fetal calf serum, gentamicin, TRIzol, CountBright™ absolute counting beads, Fast SYBR^®^ Green Master Mix, M-PER mammalian protein extraction reagent, RIPA lysis buffer and BCA protein assay kit (Thermo Fisher, Waltham, USA); human recombinant C5a (R&D Systems); C5aR1 peptide antagonist (PMX53, Ac-Phe-cyclo [Orn-Pro-dCha-Trp-Arg]); and control peptide (random sequence) (synthesized by GenScript, Shanghai, China).

### Mice

Homozygous *C5ar1^-/-^* mice were generated by homologous recombination in embryonic stem cells (Hopken et al., 1996) (provided by Dr. Bao Lu, Harvard Medical School, Boston) and backcrossed onto the C57BL/6 (H-2b) parental strain for at least 10 generations. Wild-type littermate mice were used as controls. Female mice (8–10 weeks old) were used in all experiments. All mice were maintained in specific pathogen-free conditions. The Ethics Review Committee for Animal Experimentation at Xi’an Jiaotong University approved and oversaw all mouse experiments.

### Bacterial Strains

Cystitis isolate NU14 was provided by Scott Hultgren (Washington University School of Medicine, St. Louis, MO, USA), which is a clinical isolate of *E. coli* originally obtained from the urine of a patient with cystitis. *E. coli* J96 (serotype O4: K6), a human pyelonephritis isolate, was provided by Rodney Welch (University of Wisconsin, Madison, Wisconsin, USA).

### Induction of Acute Cystitis

A murine model of acute cystitis was induced in female mice by bladder inoculation with UPEC *via* urethra as previously described with modifications (Bishop et al., 2007). In brief, cystitis isolate NU14 was cultured at 37°C in LB–Miller broth, overnight cultures were washed and resuspended in PBS, and each mouse received 1 × 10^8^ colony-forming unit [CFU] in 50 μl PBS. Mice were killed at different time points (up to 48 h post inoculation [hpi]) for the evaluation of bladder histopathology and bacterial load. In some experiments, mice were treated with the C5aR1 peptide antagonist (PMX53, 1 mg/kg) or control peptide (random sequence), as described previously (Li et al., 2017), at 2 h before and 24 h after inoculation by intraperitoneal (i.p.) injection.

### Measurement of Bacterial Load in the Bladder

Total bacterial load in bladder tissue was determined by bacterial plate count assay as previously described, with modifications (Hagberg et al., 1983; Choudhry et al., 2016). In brief, bladder tissue was weighed and subsequently homogenized in 1 ml of 0.1% Triton X-100 in PBS. Fifty microliters of a serial dilution of homogenates was plated on duplicate CLED plates. After incubation of plates for 24 h at 37°C, bacterial CFU on the plates were manually counted and expressed as average CFU per gram of bladder tissue.

### Assessment of Bladder Histopathology

Bladders were fixed in a solution of 4% formalin in PBS for 24 h and embedded in paraffin. Sections (4 µm) were stained with hematoxylin and eosin (H&E). The severity of bladder histopathology (i.e., epithelial damage/necrosis, edema, hemorrhage, and neutrophil infiltration) was graded using a 6-point scale as described previously (Garcia et al., 2013), in which 0, no lesions; 1, mild; 2, minimal; 3, moderate; 4, marked; and 5, severe. The assessment was performed in a blinded fashion by 2 persons. Two to 3 bladder sections of each mouse were viewed and are presented as an average score.

### Immunohistochemistry

To detect C5aR1 expression, frozen sections (4 µm) from normal or infected mouse bladder were stained with the anti-mouse C5aR1 monoclonal antibody at 4°C overnight and followed by Alex Fluor 488 labeled donkey anti-goat IgG, DAPI (for detection of nuclei). Sections were viewed and imaged with the confocal laser microscope system (Leica TCS SP8). *C5ar1^-/-^* mouse bladder sections were used as control. Indirect immunohistochemical staining for neutrophil was performed using the rat anti-mouse Ly6G antibody and the HRP-conjugated rabbit anti-rat polyclonal antibody. Stained bladder sections were visualized under light microscopy (Nikon 50i) and photographed at ×400 magnifications. Ly6G^+^ cells were quantified by counting the number of positively stained cells and expressed as number of Ly6G^+^ cells per field at ×400 magnification. Three to four viewing fields randomly selected from each bladder section were examined, and the average number is presented.

### Assessment of Bacterial Colonization in Bladder Tissue

For labeling bacteria, overnight cultures of NU14 were washed and resuspended in PBS (10^9^ CFU/ml). Tetramethylrhodamine (TRITC) was added to a final concentration of 1 mg/ml and incubated for 3 h with gentle shaking in the dark. Bacteria were sufficiently washed to remove unbound TRITC. For detection of bacterial colonization in bladder sections, the labeled NU14 (5 × 10^8^ CFU, in 50 μl PBS) were injected into the bladder per urethra. Mice were killed at 3 hpi; frozen sections of infected bladders were stained with DAPI and cytokeratin (for identification of bladder structure) and imaged with the confocal laser scanning microscope system (Leica TCS SP8). Bacterial colonies were counted at ×200 magnification, and results were expressed as number of colonies per field. Three viewing fields randomly selected from epithelium areas within each bladder were examined.

### Cell Culture

The J82 human bladder cell line was obtained from the American Type Culture Collection (ATCC, Manassas, VA, USA). Cells were cultured in Eagle’s Minimum Essential Medium supplemented with 10% heat-inactivated fetal bovine serum (FBS), 100 U/ml penicillin, and 100 μg/ml streptomycin at 37°C and 5% CO_2_ atmosphere of a standard incubator. Cell cultures used for experiments were limited to 3–6 passages, and the cell morphology was regularly checked.

### Assessment of Mannosyl Residue Expression in Bladder Tissue and Cultured J82 Cells

Fluorescein-labeled Galanthus nivalis lectin (GNL) which binds to [α-1,3] mannose residues (Man) was used to detect Man expression in bladder tissue and cultured J82 cells. For bladder tissue, frozen sections (4 µm) from normal or infected mouse bladder were stained with fluorescein-labeled GNL (20 μg/ml in PBS) and DAPI for 30 min and then viewed and imaged with a confocal laser scanning microscope system (Leica TCS SP8). The percentage of positive staining area in each image under ×200 magnification was calculated by using ImageJ software v1.41 (National Institutes of Health, USA). Three viewing fields for each bladder were examined. For J82 cells, cells grown on the coverslips were pretreated with C5a for 24 h were fixed in 4% paraformaldehyde and stained with fluorescein-labeled GNL and DAPI. Images were taken with the Leica SP8 system. Three viewing fields randomly selected from each coverslip were examined.

### Assessment of Bacterial Binding and Internalization by Bacterial Plate Count Assay

The assays were performed as previously described with modifications (Li et al., 2006). In brief, J82 cells were seeded onto 24-well plates at a density of 1 × 10^5^ cells/well and further cultured for 24 h. The cells were then incubated with NU14 E coli (1 × 10^6^ CFU/well, at a multiplicity of infection [M.O.I] of 10) for 1 h at 37°C with 5% CO_2_. Cells were washed four times with sterile PBS to remove unattached bacteria (determined by plating the final wash on CLED agar plates with no colonies grown up). In order to lyse the cells, 500 μl sterile H_2_O was added to each well and incubated for 15 min. The cell lysate was collected, diluted seriously (1:10 and 1:100), and plated onto CLED agar plates. The plates were incubated at 37°C for 24 h. To assess the number of internalized bacteria, J82 cells after incubation were washed and then incubated for 30 min in medium containing 50 μg/ml gentamicin to kill extracellular bacteria. The cells were then washed with sterile PBS for three times, lysed with sterile H_2_O. The cell lysate was collected, diluted (1:10), and plated onto CLED agar plates. The plates were incubated at 37°C for 24 h. Colonies formed on agar plates were manually counted and expressed as CFU/mL. In some experiments, J82 cells were cultured with or without C5a for 24 h and subjected to binding and internalization assay.

### Assessment of Bacterial Adhesion to Cultured J82 Cells by Fluorescence Microscopy

J82 cell monolayers grown on coverslips in 24-well plates were incubated with TRITC-conjugated NU14 (1 × 10^6^ CFU/well) for 1 h at 37°C. Cells were vigorously washed to remove unattached bacteria and stained with DAPI and fluorescein-labeled GNL, and then viewed and imaged with the Leica SP8 system. Bound bacteria and cell numbers were counted at ×200 magnification, and results are expressed as number of bacteria per 10^3^ tubule cells. Three viewing fields randomly selected from each coverslip were examined, and the average number is presented (Li et al., 2017).

### Western Blotting

J82 cell lysate was prepared using M-PER mammalian protein extraction reagent containing proteinase on ice. Supernatants of cell lysates were collected after centrifugation at 14,000g at 4°C for 15 min. Protein concentrations were determined by the BCA protein assay kit according to the manufacturer’s instruction. Equal amounts of protein (30 g per lane) were subjected to SDS-PAGE electrophoresis. After separation, the proteins were transferred from the gel onto PVDF membranes. The membranes were incubated with primary antibodies for overnight at 4°C followed by incubation with the HRP-conjugated secondary antibody for 1 h. Protein bands were visualized by Amersham ECL Select™ detection reagent (GE Healthcare Life Sciences, USA). Quantification of protein bands on the gel was performed by measuring the intensity of individual bands using ImageJ software. The relative amounts of phosphorylated-ERK1/2 and -IκB were generated by normalization to the total protein of respective molecules.

### Quantitative and Conventional RT-PCR

Total RNA was purified from bladder tissue or J82 cells using TRIzol reagent, followed by cDNA synthesis. To exclude genomic DNA contamination, DNase was used before reverse transcription. The conventional PCR consisted of 35 cycles of 1 min at 94°C, 1 min at 60°C, and 30 s at 72°C. Real-time PCR was performed with Fast SYBR^®^ Green Master Mix on a Step One™ Real-time PCR instrument (Thermo Fisher, Waltham, MA, USA). The 2^-ΔΔCt^ method with normalization to 18S and controls was used for calculation (Livak and Schmittgen, 2001). The controls were normal bladder tissues or J82 cell only. The information for primer sequences is given in **Table 1**.

**Table 1:** .

		

### Statistical Analysis

Data are shown as mean ± SEM or the readout of individual mice. The Mann–Whitney test (for CFU) or unpaired t test was used to compare the means of two groups. One-way or two-way ANOVA was used to compare the means of more than two independent groups. All the analyses were performed using GraphPad Prism 8 Software (GraphPad Software, La Jolla, CA, USA). *p* < 0.05 was considered to be significant.

## Results

### Expression of C5aR1 in Urinary Bladder and Elevation of Urinary C5a Following Inoculation of the Bladder With UPEC

**Figure 1:**
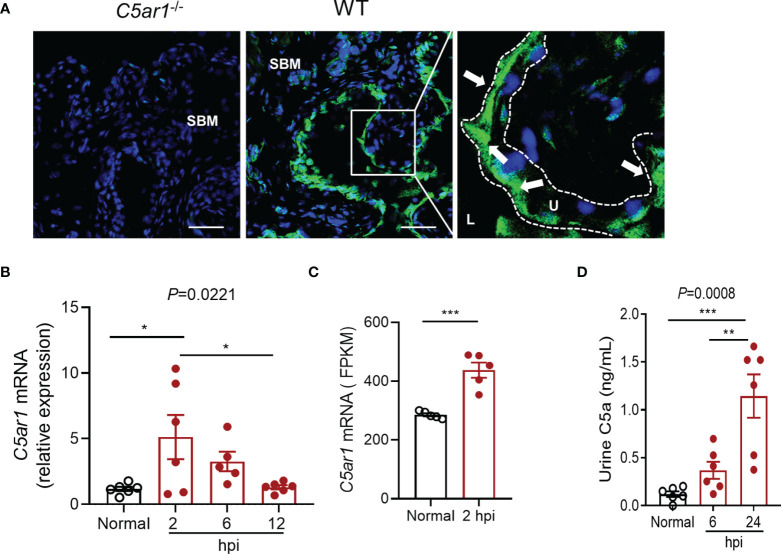
Expression of C5aR1 in urinary bladder and elevation of urinary C5a following inoculation of the bladder with UPEC. **(A)** C5aR1 expression in WT bladder tissue determined by immunohistochemistry. Bladder tissue from *C5ar1^-/-^* mice was used as negative control. Scale bars: 50 μm. Boxed regions correspond to the next (right) images. Arrows indicate positive C5aR1 staining. L, lumen. U, urothelium. SBM, submucosa. A representative of 3 experiments is shown. **(B)** Relative mRNA levels of C5ar1 in normal and infected bladder tissues determined by RT-qPCR. Data were analyzed by one-way ANOVA with Tukey’s multiple-comparison test (n = 5–6 mice/group). **(C)** Transcriptomic analysis of *C5ar1* mRNA expression in bladder tissue from B6 normal and infected mice (at 2 hpi with 1 × 10^9^ CFT073) using a dataset (GSE 33210) obtained from the NCBI GEO public database. Data were analyzed by unpaired t test (n = 5 mice/group). **(D)** C5a levels in urine samples of normal and infected mice determined by ELISA. Data were analyzed by one-way ANOVA with Tukey’s multiple-comparison test (n = 6 mice/group). *p < 0.05; **p < 0.01; ***p < 0.001. FPKM, Fragments Per Kilobase of exon model per Million mapped fragments.

The expression of C5aR1 by the urinary bladder has been reported in human previously (Zahedi et al., 2000); however, little is unknown in mice. We therefore first examined C5aR1 expression in mouse urinary bladder by immunohistochemistry and RT-PCR. Immunohistochemistry showed that positive staining of C5aR1 was mainly present in uroepithelial cells and less on the cells in submucosal space in normal WT mice, which was absent in the bladder of *C5ar1^-/-^* mice (**Figure 1A**). RT-PCR showed that *C5ar1* mRNA expression was readily detected in normal mice and upregulated following the inoculation with UPEC. A significant upregulation was observed at 2 hpi; the upregulation was declined at 6 and 12 hpi, possibly reflecting the exfoliation of bladder transitional epithelial cells following the infection (**Figure 1B**). In addition, we analyzed *C5ar1* mRNA expression in bladder tissue from B6 normal and infected mice (at 2 hpi with 1 × 10^9^ CFT073) using a dataset (GSE 33210) obtained from the NCBI GEO public database (Dulle et al., 2012). Results showed that *C5ar1* mRNA expression levels in infected bladder tissue were significantly elevated compared to normal bladder tissue (**Figure 1C**), which is consistent with our finding in RT-qPCR. We also evaluated whether C5a is present in urine and whether urinary C5a levels were altered during cystitis. ELISA showed that C5a was readily detected in normal urine and the levels were significantly elevated at 6 and 24 hpi compared with uninfected urine (**Figure 1D**).

These results demonstrate the abundant expression of C5aR1 in uroepithelial cells, upregulation of C5aR1 mRNA expression, and elevation of urinary C5a levels following the infection, which suggests that C5a/C5aR1 play roles in acute cystitis.

### C5aR1 Deficiency Protects Mice From Acute Cystitis

**Figure 2:**
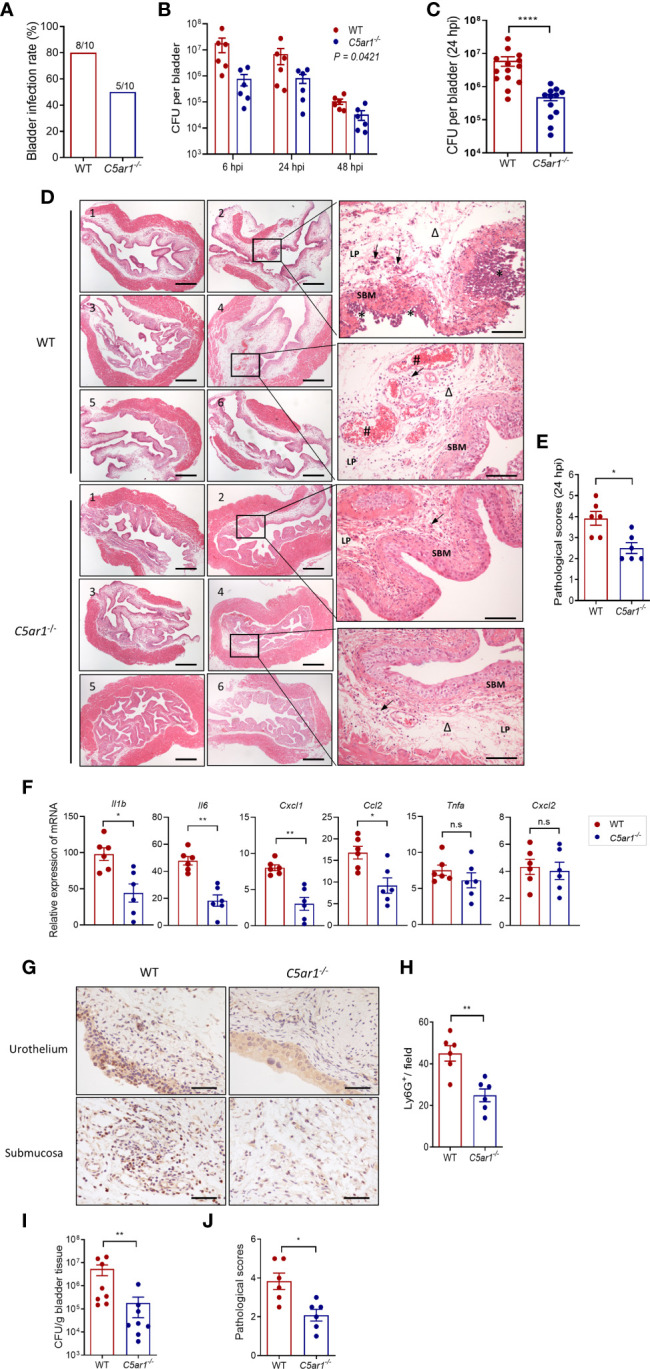
C5aR1 deficiency protects mice from acute cystitis. **(A)** Bladder infection rate of WT and *C5ar1^-/-^* mice at 6 hpi with low dose of NU14 (2.5 × 10^3^ per mouse), determined by bacterial plate count assay (n = 10 mice/group). **(B)** Bacterial loads in bladder tissues of WT and *C5ar1^-/-^* mice at 6, 24, and 48 hpi with high dose of NU14 (1 × 10^8^ per mouse). Data were analyzed by two-way ANOVA (n = 6 mice/group). **(C)** Bacterial loads in bladder tissues of WT and *C5ar1^-/-^* mice at 24 hpi (n = 12–14 mice/group, including 6 mice/group presented in B and 8 mice/(WT) group and 6 mine/(*C5ar1^-/-^*) group from additional two sets of experiments). **(D)** Representative images of H&E staining of bladder sections of WT and *C5ar1^-/-^* mice at 24 hpi. Left and middle panel: cross sections of the whole bladder at ×40. Scale bars, 500 µm. Right panel: sections of bladder tissue photographed at ×200. Scale bars, 100 µm. Symbols: triangles indicate edema; stars indicate epithelial damage; arrows indicate infiltrating cells; # indicate hemorrhage. SBM, submucosa; LP, lamina propria. **(E)** Histological scores of bladder sections of WT and *C5ar1^-/-^* mice at 24 hpi (n = 6 mice/group). **(F)** Relative mRNA levels of proinflammatory mediators in infected bladder of WT and *C5ar1^-/-^* mice at 24 hpi determined by RT-qPCR (n = 6 mice/group). **(G)** Detection of Ly6G^+^ neutrophils in the urothelium and mucosa of *WT* and *C5ar1^-/-^* mice at 24 hpi by immunohistochemistry at ×400. Scale bars, 50 µm. **(H)** Quantifications of Ly6G^+^ cells in bladder of WT and *C5ar1^-/-^* mice; results were expressed as number of Ly6G^+^ cell per filed (×400 magnification) (n = 6 mice/group; n.s. represent no significance). **(I)** Bacterial loads in bladder of WT and *C5ar1^-/-^* mice at 24 h post-inoculation with J96, determined by CFU assay (n = 8 mice/group). **(J)** Histological scores of bladder sections of WT and *C5ar1^-/-^* mice with J96 at 24 hpi (n = 6 mice/group). **(C–J)** Data were expressed as mean ± SEM and analyzed by unpaired t test. *p < 0.05; **p < 0.005; ****p < 0.0001. Each dot represents an individual mouse.

To determine the role of C5aR1 in acute cystitis, we induced the infection in WT and *C5ar1^-/-^* mice and assessed the bladder infection rate and severity of bladder infection. Bladder infection was defined by detection of bacteria in bladder tissue determined by plate count assay. The bladder infection rate was assessed at 6 hpi with a low dose of UPEC (NU14 strain, 2.5 × 10^3^ CFU per mouse). *C5ar1^-/-^* mice displayed a lower bladder infection rate compared with WT mice (50% vs. 80%) (**Figure 2A**). The severity of bladder infection was evaluated by bacterial load, histopathology, neutrophil infiltration, and tissue inflammatory mediator expression at different time points after inoculation with a high dose of UPEC (NU14 strain, 1 × 10^8^ CFU per mouse). Compared with WT mice, *C5ar1^-/-^* mice had significantly lower bacterial load in the bladder at 6, 24, and 48 hpi, assessed by plate count assay (**Figures 2B, C**). Histopathological analysis showed that compared with WT mice, *C5ar1^-/-^* mice had less severe bladder lesions (i.e., cellular infiltration, edema, hemorrhage, uroepithelium disruption) (24 hpi) (**Figures 2D, E**). RT-PCR showed that bladder mRNA levels of several key proinflammatory cytokines/chemokines (i.e., *Il6, Ilb, Cxcl1, Ccl2*), but not *Tnfα*, *Cxcl2* were significantly reduced in *C5ar1^-/-^* mice compared to WT mice (24 hpi) (**Figure 2F**). Immunohistochemistry analysis for neutrophil infiltration showed that the number of Ly6G^+^ cells in the bladder was lower in *C5ar1^-/-^* mice than in WT mice (24 hpi) (**Figures 2G, H**). In addition to the UPEC strain (NU14) mentioned above, an additional set of experiments were performed using pyelonephritic strain J96 which expresses both type 1 and P fimbriae; similar results were obtained showing that bladder bacterial load and tissue injury were significantly reduced in *C5ar1^-/-^* mice compared with WT mice at 24 hpi with J96 (1 × 10^8^ CFU per mouse) (**Figures 2I, J**).

Collectively, these results demonstrate that C5aR deficiency reduces bladder infection rate and leads to reduced bladder bacterial load (with up to 1.5 log reduction) and less severe tissue injury, thus supporting a pathogenic role for C5aR1 in acute cystitis.

### C5aR1 Deficiency Reduces Adhesion of UPEC to and Mannose Expression in the Bladder Epithelium

**Figure 3:**
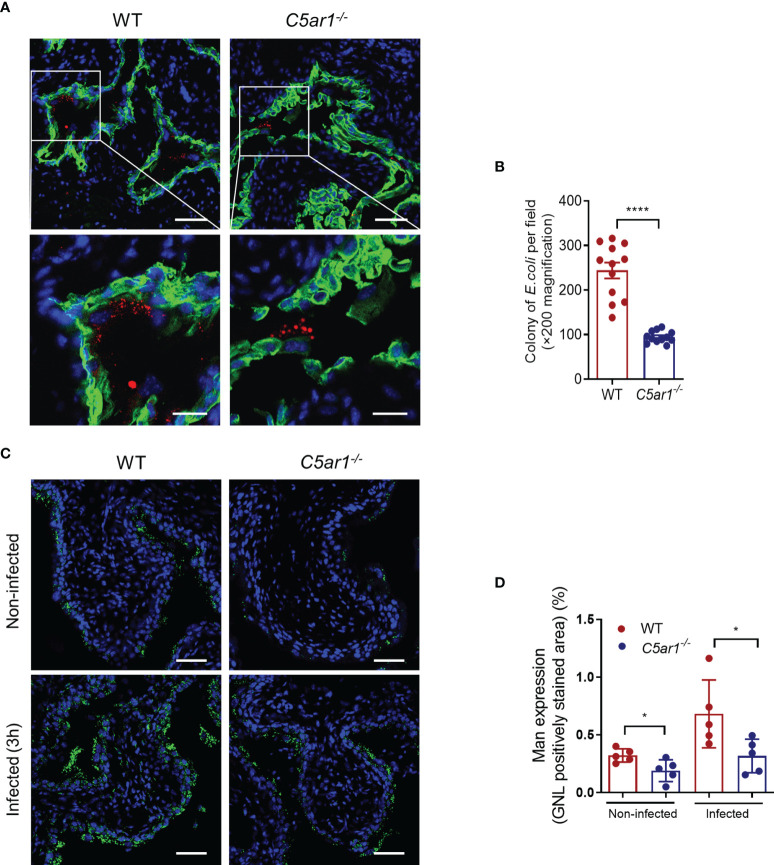
C5aR1 deficiency reduces adhesion of UPEC to and mannosyl residues expression in the bladder epithelium. **(A)** Representative fluorescence microscopy images showing early bacterial colonization of bladder epithelium in WT and *C5ar1^-/-^* mice at 6 hpi. TRITC-labeled NU14 (red), cytokeratin (green), and nuclear marker DAPI (blue) are shown. Boxed regions correspond to the below images. Scale bars, 50 μm. **(B)** Quantification of bacterial colonies in the urothelium corresponding to the WT and *C5ar1^-/-^* mice in **(A)**. Data were analyzed by unpaired test (n = 12 viewing fields from 4 mice/group). ****p < 0.001. **(C)** Representative images of mannosyl residues (Man) expression detected by fluorescein-labeled GNL (green) in bladder sections of WT and *C5ar1^-/-^* mice (i.e., non-infected and infected with NU14 at 3 hpi). Scale bars, 50 μm. **(D)** Quantification of Man expression in the transitional uroepithelium corresponding to the WT and *C5ar1^-/-^* mice in **(C)**. Data were analyzed by unpaired t test, n = 5 mice/group. *p < 0.05.

Microbial adhesion is a critical step for infection and colonization of the host. We therefore sought to assess the impact of C5aR1 deficiency on UPEC adhesion to the bladder epithelium. Confocal microscopy analysis was performed in bladder tissues of WT and *C5ar1^-/-^* mice at 6 h following inoculation with fluorescent-labeled UPEC (NU14 strain). There were significantly fewer colonies in the bladder epithelium of *C5ar1^-/-^* mice compared with WT mice (**Figures 3A, B**). It has been shown that the interaction of type 1 fimbriae and mannose-containing host receptors in the uroepithelium represents an important mechanism of bladder infection (Langermann et al., 2000; Spaulding et al., 2017; Klein and Hultgren, 2020). Furthermore, in our recent studies we have shown that both Man and C5aR1 were predominantly detected on the luminal surface of renal tubules; a colocalization, albeit partial, was observed between C5aR1 and Man, suggesting that C5aR1 is a mannose-containing receptor (Li et al., 2017). In addition, Man expression in the renal tubular epithelium upon infection was significantly lower in *C5ar1^-/-^* mice compared with WT mice, and Man expression in murine and human RTEC was upregulated by C5a stimulation, suggesting a role of C5aR1 in regulation Man expression in renal tubular epithelial cells (Li et al., 2017; Song et al., 2018). These promoted us to investigate whether C5aR1 deficiency has impact on Man expression in the bladder epithelium. Fluorescein-labeled GNL was applied to detect Man in bladder tissues of uninfected and infected (3 hpi) WT and *C5ar1^-/-^* mice and followed by confocal microscopy analysis. GNL staining was increased in the bladder epithelium of infected mice compared with uninfected mice for both WT and *C5ar1^-/-^* mice. There was significantly less GNL staining in the bladder epithelium of *C5ar1^-/-^* mice compared with WT mice for both uninfected and infected mice (**Figures 3C, D**). These results demonstrate that C5aR1 deficiency reduces adhesion of UPEC to and mannose expression in the bladder epithelium.

### C5a Stimulation Upregulates Mannosyl Residue Expression and Enhances UPEC Adhesion/Invasion in Bladder Epithelial Cells *In Vitro*

**Figure 4:**
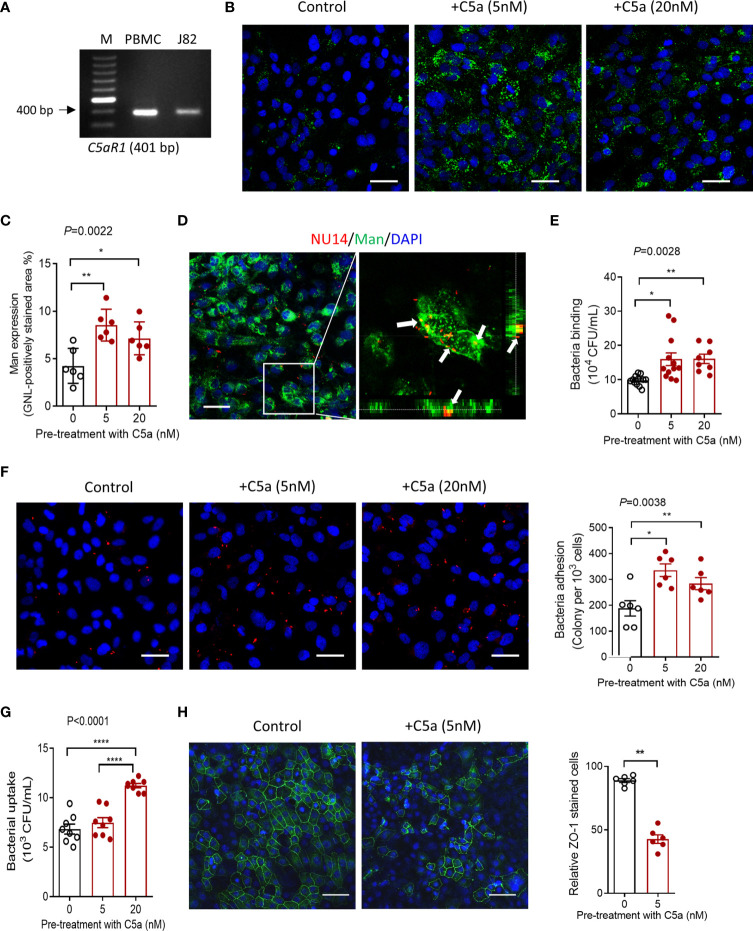
C5a stimulation upregulates mannosyl residue expression and enhances UPEC adhesion/invasion in bladder epithelial cells *in vitro*. **(A)** The agarose gel of conventional PCR showing the detection of C5aR1 in cultured human J82 cells. Human PBMC was used as positive control. **(B–H)** J82 cells were incubated with or without C5a (5, 20 nM) for 24 h, then incubated with or without NU14 (bacteria: 1 × 10^6^) for 1 h at 37°C. **(B)** Representative fluorescence images of mannosyl expression in non-treated (control) and C5a pretreated J82 cells. Man (green) detected by fluoresce-labeled GNL and DAPI (blue) are shown. Scale bars, 50 μm. **(C)** Quantification of Man expression, shown as fold change in GNL-positively stained area corresponding to the images shown in **(B)**. Data were analyzed by one-way ANOVA with Tukey’s multiple-comparison test (n = 6 viewing fields from 2 coverslips per group) and representative of 3 independent experiments. **(D)** Confocal microscopic images of bound bacteria in J82 cells that had been incubated with TRITC-labeled NU14 for 1 h. E. coli (red), mannosyl residue (green), and DAPI (blue) are shown. Boxed region in the left image corresponds to the right image. Scale bars, 50 μm. Right image, cross-sectional views (bottom and side panel) of J82 cells, Man and bacteria, demonstrating colocalization of Man and NU14 at cell surface. **(E)** Binding of NU14 to J82, evaluated by bacterial plate count assay. Data were analyzed by one-way ANOVA with Tukey’s multiple-comparison test (n = 8–12 wells per group, pooled from 2 individual experiment). **(F)** Representative fluorescence images of bound bacteria in J82 cells that had been pretreated with or without C5a for 24 h, then incubated with TRITC-labeled NU14 (red) for 1 h, DAPI (blue) are shown. Scale bars, 50 μm. Quantification of bound bacteria corresponding to the images is shown on the right panel. Data were analyzed by one-way ANOVA with Tukey’s multiple-comparison test (n = 6 images/group, pooled from 2 coverslips) and representative of 3 independent experiments. **(G)** Quantification of bacteria uptake by J82 cells, evaluated by bacterial plate count assay. Data were analyzed by One-way ANOVA with Tukey’s multiple-comparison test (n = 8 wells per group, pooled from 2 individual experiments). **(H)** Representative fluorescence images of ZO-1 (green) staining in J82 cells, incubated with C5a (5 nM) or control for 24 h, 1 h after incubation with bacteria. Scale bars, 50 μm. Quantification of ZO-1-positive cells is shown on the right panel. Data were analyzed by unpaired t test (n = 6 images/group) and representative of 2 independent experiments. *p < 0.05; **p < 0.01; ****p < 0.0001.

Having observed that C5aR1 was mainly detected in the bladder epithelium, and Man expression and UPEC adhesion in the bladder epithelium were dependent on C5aR1 (**Figures 1**, **3**), next, we sought to assess whether the C5a/C5aR1 interaction has effects on Man expression and UPEC adhesion in cultured human bladder epithelial cells (J82 cells). We first confirmed that C5aR1 is expressed in the cells by RT-PCR using human peripheral blood mononuclear cells (hPBMC) as positive control (**Figure 4A**). We then assessed the effects of C5a stimulation on Man expression in the epithelial cells. C5a (5, 20 nM) stimulation significantly upregulated Man expression on the cell surface, determined by GNL staining and fluorescence microscopy (**Figures 4B, C**). UPEC binding to bladder epithelial cells and its association with Man on the cell surface were further confirmed by confocal microscopy (**Figure 4D**). Next, we tested if the C5a-mediated modulation in J82 cells (e.g., activation, Man expression) can influence UPEC invasion/invasion. UPEC adhesion was assessed by CFU and fluorescence microscopy following incubation of the epithelial cells with unlabeled or fluorescence-labeled UPEC. Pretreatment of the epithelial cells with C5a (5, 20 nM) for 24 h significantly enhanced UPEC adhesion to epithelial cells (**Figures 4E, F**). UPEC invasion was assessed by CFU of internalized UPEC, and disruption of epithelial barrier integrity was detected using fluorescence microscopy. Pretreatment of the epithelial cells with C5a (5, 20 nM) for 24 h significantly increased UPEC internalization (**Figure 4G**) and epithelial barrier damage (**Figure 4H**). Collectively, these results demonstrate the effects of C5a stimulation on upregulation of mannose expression and UPEC adhesion/invasion in human bladder epithelial cells, which aligns with *in vitro* observation on C5aR1-dependent upregulation of mannose expression and UPEC adhesion in bladder epithelium, suggesting that those processes were driven by the C5a/C5aR1 axis.

### C5a Activates ERK1/2/IκB Signaling and Upregulates Proinflammatory Cytokine Production in Human Bladder Epithelial Cells *In Vitro*

**Figure 5:**
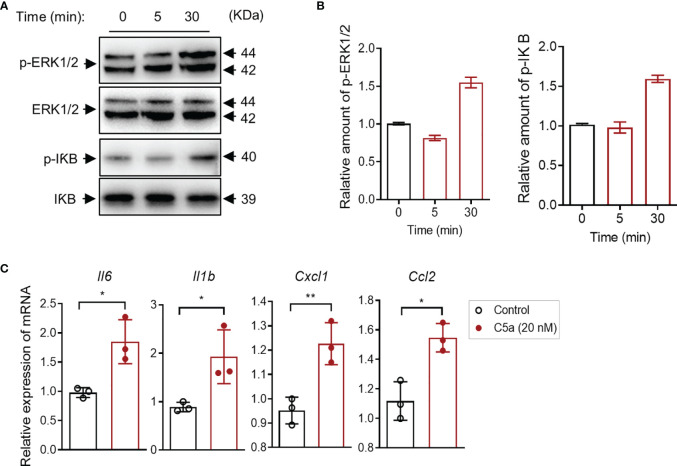
C5a activates ERK1/2/IκB signaling and upregulates proinflammatory cytokine production in human bladder epithelial cells *in vitro*. **(A)** Western blot analysis for ERK1/2 and IκB phosphorylation in J82 cells after C5a (20 nM) stimulation for up to 30 min. In each set of blots, the top row of bands corresponds to incubating membrane with appropriate anti-phospho-antibody and the bottom row of bands corresponds to incubating membrane with appropriate total antibody. **(B)** Relative amounts of protein phosphorylation are shown in the right panel of each set of blots. A representative of two independent experiments is shown. **(C)** J82 cells were incubated with C5a (0, 20 nM) for 6 h. Relative mRNA levels of pro-inflammatory mediators in the cell, were determined by RT-PCR (n = 3/group). Data were analyzed by unpaired t test. *p < 0.05, **p < 0.01.

It has been shown that the C5a/C5aR1 interaction induces intracellular signal transduction and regulates cytokine/chemokine production in various types of cells. Little is known in bladder epithelial cells. Given that C5aR1 is expressed in bladder epithelial cells, we reasoned that C5a may have effects on intracellular signal transduction and cytokine production through interaction with C5aR1 in the cells. To assess the effects of C5a on intracellular signal transduction, we performed Western blotting in bladder epithelial cells following C5a stimulation (for 0, 5, and 30 min) to examine the changes in phosphorylation levels of ERK1/2 and IκB (which plays a critical role in mediating pro-inflammatory responses). C5a (20 nM) stimulation for 30 min clearly increased the phosphorylation of ERK1/2 and IκB in the cells, but the stimulation for 5 min did not show a significant increase in the phosphorylation (**Figures 5A, B**). To assess the effects of C5a on proinflammatory cytokine production, we performed RT-PCR in the cells following C5a stimulation. C5a (20 nM) stimulation for 6 h significantly increased the gene expression of a set of pro-inflammatory mediators (i.e., *Il6, Il1b, Cxcl1, Ccl2*) in the cells (**Figure 5C**). These results demonstrate that C5a can induce a pro-inflammatory signal and upregulate proinflammatory mediator production in bladder epithelial cells.

### Pharmacological Administration of C5aR1 Antagonist Protects Mice From UPEC-Induced Acute Cystitis

**Figure 6:**
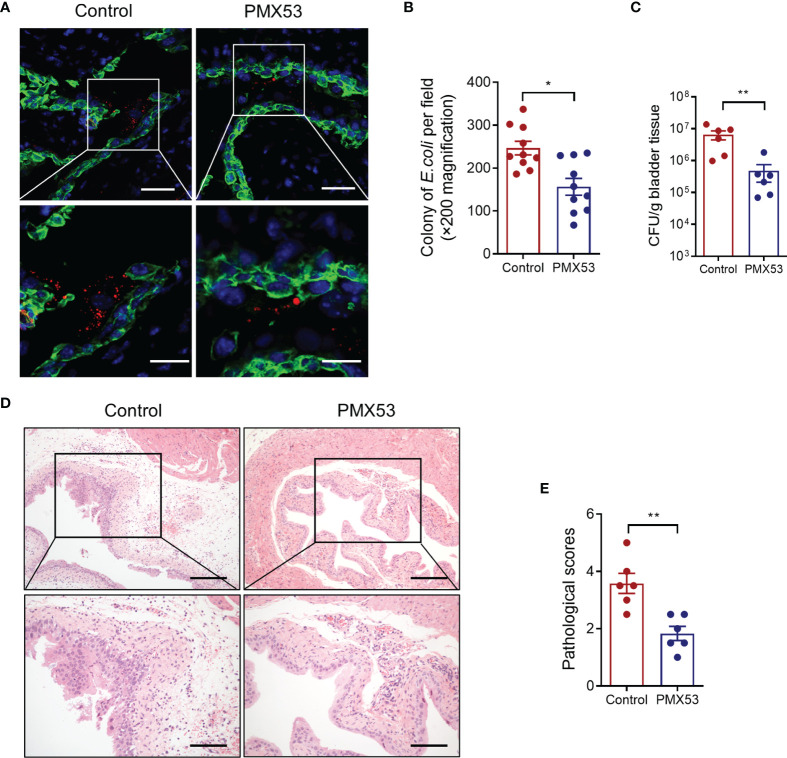
Pharmacological administration of C5aR1 antagonist protects mice from UPEC-induced acute cystitis. **(A)** Representative images showing early bacterial colonization of bladder epithelium in control or PMX53-treated mice (6 hpi). TRITC-labeled NU14 (red), cytokeratin (green), and nuclear marker DAPI (blue) are shown. Boxed regions corresponding to the below images. Scale bars, 50 μm. **(B)** Quantification of bacterial colonies in bladder urothelium corresponding to the control and PMX53-treated mice in **(A)**. **(C)** Bacterial loads in bladder tissues of control and PMX53-treated mice were also determined by bacterial plate count assay at 24 hpi (n = 6/group). **(D)** Representative images of H&E staining of bladder sections of control and PMX53-treated mice at 24 hpi. Scale bars, 200 μm. **(E)** Histological scores of bladder sections of control and PMX53-treated mice in **(D)** (n = 6/group). Data were analyzed by the unpaired t test. *p < 0.05; **p < 0.01.

C5aR antagonists PMX53 have been extensively used in preclinical studies in various species, including mice (Kumar et al., 2020). Our previous study has also shown that pharmacological administration of the C5aR1 antagonist protected mice from UPEC-induced acute kidney infection (Choudhry et al., 2016; Li et al., 2017). We therefore sought to assess whether blockade of C5aR1 could provide protection against acute bladder infection. We treated WT mice with PMX53 or control peptide (1 mg/kg) intraperitoneally 2 h before infection and 6 hpi after inoculation with UPEC. Mice were killed at 6 or 24 hpi. Early bacterial colonization, tissue bacterial load, and histopathology were analyzed. Early bacterial colonization was assessed at 6 hpi by fluorescent microscopy analysis; bladder bacterial load and histopathology changes were assessed at 24 hpi by CFU plate assay and microscopy analysis, respectively. As we expected, PMX53-treated mice had significantly reduced early bacterial colonization (**Figures 6A, B**), bladder bacterial load (with >1 log reduction) (**Figure 6C**), and bladder histopathological lesions (**Figures 6D, E**), compared with the control group. Taken together, these results demonstrate that blockade of C5aR1 provides protection against acute bladder infection.

## Discussion

Our previous work using a murine model of pyelonephritis has demonstrated that the C5a/C5aR1 interaction plays a pathogenic role in acute and chronic kidney infection (Choudhry et al., 2016; Li et al., 2017; Klein and Hultgren, 2020). In the present study, we extended our previous work to the bladder infection. By using a murine model of cystitis combining gene ablation and pharmacological blockade of C5aR1, we demonstrate that the C5a/C5aR1 axis also plays a pathogenic role in bladder infection. A mechanistic investigation suggests that C5a/C5aR1 axis-mediated upregulation of Man expression, enhancement of bacterial adhesion/colonization, and excessive inflammatory responses contribute to acute bladder infection. The findings of this study improve our understanding of the pathogenesis of bladder infection with therapeutic implications for UTI.

Cystitis is one of the most common human infectious diseases, mainly caused by UPEC. Despite the importance of the innate immune system in fighting UPEC infection, the pathogenesis of UTI is complex and can be influenced both by properties of the infecting pathogens and by host responses to pathogens, in addition to other factors such as anatomical abnormality (Abraham and Miao, 2015; Klein and Hultgren, 2020). Therefore, besides the properties of UPEC, the host immune response to pathogens has a big impact on the pathogenesis of UTI. Although innate immune responses play essential roles in the first line of host defense against pathogens, they also cause harm when present in excess or are dysregulated. For example, in acute conditions, uroepithelial cells and inflammatory cells, in response to UPEC stimulation, produce a number of pro-inflammatory mediators (e.g., IL-6, TNF-α, and IL-8), which (if present in excess) cause epithelial inflammation/damage, allowing bacteria to enter the underlying tissue (Godaly et al., 2015). In addition, if the activation of neutrophils is not tightly regulated, the reactive oxygen species and cytotoxic enzymes and ingested bacteria could be released into the surrounding area causing tissue destruction and pathogen dissemination (Kennedy and DeLeo, 2009). Previous studies in acute pyelonephritis have found that, paradoxically, *Tlr4^-/-^*, *Il1b^-/-^*, or *C3^-/-^* mice or mice receiving anti-inflammatory mediator cyclic AMP had less severe acute kidney infection and inflammation, suggesting that innate immune responses driven by TLR4 signaling, pro-inflammatory cytokine IL-1β, or complement effector molecules are harmful, rather than beneficial, for the host (Springall et al., 2001; Yadav et al., 2010; Wei et al., 2015). With regard to the role of C5aR1 in UTI, it is worth noting a number of observations from our own and others’ published work as well as the work presented in this manuscript: i) most human UPEC strains are resistant to complement-mediated killing (Li et al., 2009; Bjanes and Nizet, 2021), but on the other hand, excessive C5a can be generated by persistent activation of complement during infection and detected in the urine of infected mice (Li et al., 2017); ii) C5aR1 is expressed in uroepithelial cells including renal tubular epithelial cells and transitional epithelium of the ureter and urinary bladder (Zahedi et al., 2000), in addition to the migrated leukocytes; and iii) C5aR1 and TLR4 cross talk upon pathogen invasion has synergistic effects on proinflammatory cytokine production (Zhang et al., 2007). Therefore, although C5a/C5aR1 interaction-mediated inflammatory response is generally required for fight infection, in UTI, the cross talk between C5aR1 and TLR4-mediated excessive inflammatory responses, together with the lack of complement-mediated direct killing, will lead to overactivation of immune response which is harmful rather than beneficial.

With regard to the possible mechanisms by which C5a/C5aR1 promotes bladder infection, one of the important findings we made is that C5a/C5aR1 has the effects of upregulating Man expression in bladder epithelial cells and promoting bacterial adhesion/colonization. The uroepithelial cell lining of the urinary tract is the first point of contact for potential pathogens. Type 1 fimbriated *E. coli* are associated with colonization of the lower urinary tract. Binding of FimH, a mannose-specific lectin existing at the tip of type 1 fimbriae of UPEC, to high mannose-type glycoproteins (e.g., Uroplakins Ia) that coat on the apical surface of bladder epithelium is the first step of UPEC infection (Wu et al., 1996; Xie et al., 2006). In our *in vivo* study, we first assessed the possibility that the C5a/C5aR1 axis contributes to bladder infection through upregulating Man expression in the urinary bladder epithelium. Firstly, GNL staining of bladder tissue demonstrated that C5aR1 deficiency is associated with reduced levels of Man expression on the luminal surface of the bladder epithelium in both non-infected and infected mice, reflecting a requirement for C5a/C5aR1 interactions for Man expression in both normal and pathogenic conditions. Given the expression of C5aR1 in the apical surface of the uroepithelium, and the upregulation of C5aR1 expression in the bladder and elevation of C5a levels in urine following the infection, it is possible that C5a/C5aR1 interactions could occur during UTI and this in turn leads to an upregulation of Man expression. Our *in vitro* findings that C5a stimulation upregulated cell surface Man expression and enhanced UPEC adhesion to human bladder J82 cell further support the notion that C5a/C5aR1 interactions mediate the upregulation of Man in the uroepithelium, which enhances Type 1 fimbriated *E. coli* adhesion/colonization. In addition, our previous study in murine kidney infection has shown a large-scale association between C5aR1 and Man on the luminal surface of the renal tract epithelium by confocal microscopy (Li et al., 2017); this, together with the fact that C5aR1 is an N-linked glycosylated protein, raises the possibility that C5aR1 itself as a mannose-containing glycoprotein may function as a carbohydrate ligand for UPEC and mediate type 1 fimbriae-dependent bacterial adhesion/colonization.

In addition to the effect of upregulating Man expression and promoting bacterial adhesion/colonization, another important finding we made is that C5a/C5aR1 has a potent effect on promoting inflammatory responses in the bladder. Following bladder inoculation, gene deletion or blockade of C5aR1 significantly reduced bladder tissue inflammation(i.e., neutrophil infiltration, bladder edema and hemorrhage, uroepithelium disruption, and upregulation of proinflammatory mediator gene expression). These findings suggest that C5aR1 plays a critical role in upregulating local inflammatory responses to UPEC, which could contribute to bladder tissue injury. In addition, C5aR1-mediated upregulation of Man expression and enhancement of bacterial adhesion/colonization and subsequent submucosal invasion could also contribute to bladder tissue injury. With regard to the cellular basis of the C5a/C5aR1 axis contributing to tissue inflammation, the role of the C5a/C5aR1 interaction in activating macrophage to produce cytokine/chemokine and its relevance to UTI have been studied in our previous studies (Choudhry et al., 2016). In the present, we examined the impact of the C5a/C5aR1 interaction on cellular proinflammatory cytokine production in bladder epithelial cells. Our *in vitro* findings that C5a stimulation mediated the activation of the ERK1/2/NF-κB signaling pathway and upregulation of gene expressions of *Il6, Il1b, Cxcl1*, and *Ccl2* by cultured bladder epithelial cells, together with the effects of the C5a/C5aR1 axis on inflammatory cells, suggest that C5aR1 on both inflammatory and renal parenchymal cells could contribute to local tissue inflammation.

In the present study, C5a concentrations at 5 and 20 nM were found effective in upregulating Man expression and cytokine and chemokine gene expression in bladder epithelial cells. Although C5a serum levels in normal conditions are very low (<1 nM), higher concentrations (up to 60 nM) were found in pathological conditions (Bengtson and Heideman, 1988; Bosmann et al., 2011). Various concentrations up to 100 nM have been used for studying the effects of C5a on diverse cellular responses in immune cells and parenchymal cells in *in vitro* experiments (Choudhry et al., 2016; Li et al., 2017; Song et al., 2018; Wood et al., 2020).

We noticed that our RT-PCR analysis in bladder tissue showed that C5aR1 difference led to downregulation of gene expressions of Il6, IL1b, Cxcl1, and Ccl2, but not Tnfa and Cxcl2. The reasons for this are unclear. One possible explanation could be that within the infected bladder, upregulation of Tnfa and Cxcl2 is predominantly dependent on TLR4 signaling, whereas upregulation of the other cytokine/chemokine genes is predominantly dependent on C5a/C5aR1 signaling or the cross talk between C5aR1 and TLR4. Another explanation could be that the gene expression levels of Tnfa and Cxcl2 may not well reflect their protein levels. Another limitation of our study is that cytokine/chemokine protein levels were not measured in the bladder tissue.

The murine model of UTI which we employed in this study was well characterized and widely used for studying acute cystitis. The model is highly relevant to human cystitis, as the infection is induced by the human UPEC strain, the route of infection resembles that of human cystitis, and the pathology is close to the human situation. The findings presented in this study, coupled with the findings of our previous studies in pyelonephritis (Choudhry et al., 2016; Li et al., 2017; Song et al., 2018), highlight pathogenic roles for C5aR1 in UTI and open up a new avenue for therapeutic targeting in human UTI, as therapeutic reagents targeting the C5a/C5aR1 axis suitable for clinical use are available (Zelek et al., 2019; Lee, 2022). The present study examined that the effect of the C5aR antagonist (PMX53) was administered *via* i.p. injection; it is unknown whether oral administration of PMX53 is effective in this model, which warrants further investigation.

In conclusion, this study clearly demonstrates a pathogenic role for the C5a/C5aR1 axis in low urinary tract infection and suggests two distinct mechanisms contributing to the pathology of acute cystitis, namely, enhancement of bacterial adhesion/colonization and excessive inflammatory responses. It improves our understanding of the mechanisms of bladder infection with therapeutic implications for UTI.

## Data Availability Statement

The original contributions presented in the study are included in the article/supplementary material. Further inquiries can be directed to the corresponding authors.

## Ethics Statement

The animal study was reviewed and approved by the Ethics Review Committee for Animal Experimentation of Xi’an Jiaotong University. Written informed consent was obtained from the owners for the participation of their animals in this study.

## Author Contributions

KYW, BC, CXW, XLY, SJZ, TYD and GXZ performed experiments. KYW, JRY and LRL analyzed data. WZ helped with experimental design, critical reading and discussion of the manuscript. JRY: Data interpretation and helped with experimental design. KYW, BC and KL wrote the manuscript. KL conceived and designed the study, and supervised the project. All authors contributed to the article and approved the submitted version.

## Funding

This work was supported by the National Natural Science Foundation of China (NSFC 81900620 to K-YW, NSFC 81970596 to KL), the Basic and Frontier Research Program of Chongqing (cstc2017jcyjBX0014 to J-RY), and the Natural Science Foundation of Shaanxi Province (2018JQ8045 to T-YD, 2020JQ536 to K-YW).

## Conflict of Interest

The authors declare that the research was conducted in the absence of any commercial or financial relationships that could be construed as a potential conflict of interest.

## Publisher’s Note

All claims expressed in this article are solely those of the authors and do not necessarily represent those of their affiliated organizations, or those of the publisher, the editors and the reviewers. Any product that may be evaluated in this article, or claim that may be made by its manufacturer, is not guaranteed or endorsed by the publisher.
